# Possible New Histological Prognostic Index for Large B-Cell Lymphoma

**DOI:** 10.3390/jcm12196324

**Published:** 2023-09-30

**Authors:** Hideaki Nitta, Haruko Takizawa, Toru Mitsumori, Hiroko Iizuka-Honma, Yoshihiko Araki, Maki Fujishiro, Shigeki Tomita, Satsuki Kishikawa, Akane Hashizume, Tomohiro Sawada, Mitsuo Okubo, Yasunobu Sekiguchi, Miki Ando, Masaaki Noguchi

**Affiliations:** 1 Department of Hematology, Juntendo University Urayasu Hospital, 2-1-1 Tomioka, Urayasu-shi 279-0021, Japan; nitta@juntendo.ac.jp (H.N.); takizawa@juntendo.ac.jp (H.T.); t.mitsumori.db@juntendo.ac.jp (T.M.); hiiduka@juntendo.ac.jp (H.I.-H.); 2 Department of Pathology and Microbiology, Division of Microbiology, Nihon University School of Medicine, Itabashi-ku, Tokyo 173-8610, Japan; yaraki@juntendo.ac.jp; 3 Institute for Environmental and Gender-Specific Medicine, Juntendo University Urayasu Hospital, Chiba 279-0021, Japan; mfujishi@juntendo.ac.jp; 4 Department of Diagnostic Pathology, Juntendo University Urayasu Hospital, Chiba 279-0021, Japan; sstomita@juntendo-urayasu.jp (S.T.); s-kishikawa@juntendo.ac.jp (S.K.); akane@juntendo-urayasu.jp (A.H.); 5 Department of Clinical Laboratory, Juntendo University Urayasu Hospital, Chiba 279-0021, Japan; sawada@juntendo-urayasu.jp; 6 Laboratory of Blood Transfusion, Juntendo University Urayasu Hospital, Chiba 279-0021, Japan; mi-okubo@juntendo.ac.jp; 7 Hematology Clinic, Saitama Cancer Center, Saitama 362-0806, Japan; yasu_sek@saitama-pho.jp; 8 Division of Hematology, Juntendo University Juntendo Hospital, 3-1-3 Hongo, Bunkyo-ku, Tokyo 113-0033, Japan; m-ando@juntendo.ac.jp

**Keywords:** large B-cell lymphoma (LBCL), immunohistochemical (IHC) staining, glucose-regulated protein 94 (GRP94), CYP3A4, multidrug resistance protein 1 (MDR1), aldo-keto reductase family 1 member C3 (AKR1C3), multidrug resistance-associated protein 1 (MRP1), P53

## Abstract

We conducted a retrospective analysis of GRP94 immunohistochemical (IHC) staining, an ER stress protein, on large B-cell lymphoma (LBCL) cells, intracellular p53, and 15 factors involved in the metabolism of the CHOP regimen: AKR1C3 (HO metabolism), CYP3A4 (CHOP metabolism), and HO efflux pumps (MDR1 and MRP1). The study subjects were 42 patients with LBCL at our hospital. The IHC staining used antibodies against the 17 factors. The odds ratios by logistic regression analysis used a dichotomous variable of CR and non-CR/relapse were statistically significant for MDR1, MRP1, and AKR1C3. The overall survival (OS) after R-CHOP was compared by the log-rank test. The four groups showed that Very good (5-year OS, 100%) consisted of four patients who showed negative IHC staining for both GRP94 and CYP3A4. Very poor (1-year OS, 0%) consisted of three patients who showed positive results in IHC for both GRP94 and CYP3A4. The remaining 35 patients comprised two subgroups: Good (5-year OS 60–80%): 15 patients who showed negative staining for both MDR1 and AKR1C3 and Poor (5-year OS, 10–20%): 20 patients who showed positive staining for either MDR, AKR1C3, MRP1, or p53. The Histological Prognostic Index (HPI) (the four groups: Very poor, Poor, Good, and Very good) is a breakthrough method for stratifying patients based on the factors involved in the development of treatment resistance.

## 1. Introduction

R-CHOP (Rituximab, Cyclophosphamide, Hydroxydaunorubicin hydrochloride, Oncovin, Prednisone) is the standard treatment regimen for LBCL, although about 40% of LBCL patients receiving R-CHOP therapy develop treatment resistance [1]. Comprehensive analyses for numerous genes have been conducted with the objective of developing strategies to overcome treatment resistance in these patients [2,3]. On the other hand, for many functional proteins involved in treatment resistance, the majority of studies have been published as one-off reports. In this study, we attempted a retrospective comprehensive immunohistochemical (IHC) analysis of 17 important functional factors that have been reported as being involved in the development of treatment resistance.

The 17 factors belonging to the following classes (1)–(4):

(1) ER stress proteins on the cellular membrane: glucose-regulated protein 94 (GRP94) in LBCL [4,5,6], GRP78 [7,8], transforming growth factor β1 (TGFβ1) [9,10], and tumor necrosis factor α1 (TNFα1) [11]. GRP94 and GRP78 are stress-inducible molecules released into the extracellular space that act to overcome various stresses in the tumor microenvironment, including hypoxia, hypoglycemia, dysregulation of homeostasis, altered cellular metabolism, and acidosis. TGFβ1 plays an important role in promoting tumor progression. TNF inhibits tumor progression.
(2) Enzymes in the cellular cytoplasm involved in anticancer drug metabolism: Aldo-keto reductase family 1 member C3 (AKR1C3) in LBCL [12,13,14,15,16], CYP3A4 [17,18], and CYP2B6 [19]. AKR1C3 is mainly found in the cytoplasm. AKR1C3 catalyzes the reduction of carbonyl groups to water-soluble alcohol groups. AKR1C3 lowers the activities of hydroxyl doxorubicin (H) and oncovin (O) (HO of CHOP) [14] and the risk of disease progression in patients with LBCL carrying AKR1C3 [12]. CYP3A4 inactivates many anticancer drugs, including all components of the CHOP regimen. CYP3A4 has been evaluated as a predictor of the tumor response to chemotherapy in patients with peripheral T-cell lymphomas [17]. Thus, the expression of these enzymes may lower the efficacy of the drugs used for treatment, resulting in drug resistance [17,18]. CYP2B6 activates cyclophosphamide [19].
(3) Anticancer drug efflux pumps on the cellular membrane: multidrug resistance protein 1 (MDR1) [20,21,22], multidrug resistance-associated protein 1 (MRP1) [23,24], and MRP4 [25]. MDR1 and MRP1 found on the cell membrane are hydroxyl doxorubicin (H) and oncovin (O) (HO of CHOP) efflux pumps. Overexpression of MDR1 and MRP1 leads to the development of drug resistance in tumors [20]. Patients with LBCL not harboring MDR1 and MRP1 have a relatively good prognosis [21].
(4) Other items include the revised International Prognostic Index (R-IPI)-poor and high-grade B-cell lymphoma (HGBCL), such as double-hit lymphoma (DHL), MYC translocation LBCL, follicular lymphoma transformation, lymphoplasmacytic lymphoma transformation, and HIV-related Burkitt lymphoma. In addition, double-expression (MYC and BCL2), p53 [26], Ki-67 [26], CD5, glutathione-S-transferase (GST) [27], the presence/absence of fibrosis, and thymidine phosphate [28] were also investigated.

The tumor expression levels of GRP94 [4,5,6], CYP3A4 [17], MDR1 [20,21,22], AKR1C3 [12,13,14,15,16], p53 [26], and MRP1 [21] were identified as being significantly associated with treatment resistance in patients with LBCL.

## 2. Material and Methods

For the patients and sample collection of patients diagnosed as having LBCL who received the initial treatment at our hospital between 2012 and 2020, 42 patients were selected as the study subjects.

Chemotherapy was started with the R-CHOP regimen in all 42 patients. Analyses of survivals (overall survival (OS) and progression-free survival (PFS)) after the initial R-CHOP therapy and in relation to the expression status of factors, etc. involved in anticancer drug metabolism were conducted by the Kaplan–Meier method and compared statistically by the log-rank test. In addition, logistic regression analysis (odds ratios) was performed using the dichotomized variables of CR and non-CR/relapse after R-CHOP. After weighting the predictive factors for the survival time, a combined multiple regression analysis yielded the following multiple regression equation to estimate the predicted survival time (month). after weighting the predictive factors for the survival time, a combined multiple regression analysis yields the multiple regression equation.

### 2.1. Immunohistochemistry

Biopsy specimens from the patients were fixed in formalin and embedded in paraffin to prepare tissue blocks, which were then sectioned and stained. The primary antibodies against the major proteins involved in anticancer drug metabolism included (1) GRP94: Proteintech (Rosemont, IL 60018, USA), clone 1H10B7 (This monoclonal antibody was generated against the N-terminal region of full-length HSP90b1.); (2) CYP3A4: Sigma-Aldrich (St. Louis, MO 63103, USA), SAB1400064 (This polyclonal antibody was generated against CYP3A4.); (3) AKR1C3: Proteintech, 11194-1-AP (This polyclonal antibody was generated against AKRC3.); (4) MDR1 (P-glycoprotein): Proteintech, 22336-1-AP (This polyclonal antibody was generated against MDR1.); (5) MRP1 (CD9): Proteintech, 60232-1-IG (This monoclonal antibody was generated against the N-terminal region of full-length MRP1.); (6) TGF beta1: Proteintech, 21898-1-AP (This polyclonal antibody was generated against the TGF-beta.); (7) GRP78: Proteintech, 66574-1-IG (This monoclonal antibody was generated against the N-terminal region of full-length GRP78.); (8) glutathione S-transferase kappa1 (GST): Proteintech, 14535-1-AP (This polyclonal antibody was generated against the GST1.); (9) thymidine phosphorylase: Abcam (Cambridge, UK), ab226917 (This polyclonal antibody was generated against thymidine phosphorylase.); (10) MRP4 (ABCC4): SANTA CRUZ BIOTECHNOLOGY (Dallas, TX 75220, USA), SC-376262 (This monoclonal antibody was generated against the N-terminal region of full-length MRP4 (amino acid 1-280).); (11) CYP2B6: LifeSpan BioSciences, Inc. (Seattle, WA 98121, USA)., LS-C352084 (This polyclonal antibody was generated against CYP2B6.); and (12) TNFα1: Sigma-Aldrich, SAB4502982 (This polyclonal antibody was generated against TNFα1.). After the immunostaining, two pathologists definitively determined the results of the IHC staining. Positive judgment criteria for IHC staining are more than 50% of tumors, and weakly positive abnormalities were considered. The concordance rate for the staining results was about 89%. In the case of disagreement on the staining result between the two pathologists, the final diagnosis was arrived at by consensus.

### 2.2. Statistical Analysis

Odds ratios were calculated by univariate logistic regression analysis using the dichotomized variable of “remission” versus “non-remission or relapse” as a dependent variable and each “poor prognostic factor” as an independent variable. Poor prognostic factors that significantly contributed to the outcomes were determined.

Then, to confirm the association between the OS and poor prognostic factors/factors involved in anticancer drug metabolism after the initial R-CHOP therapy, survival curves were plotted by the Kaplan–Meier method, and the factors significantly associated with the OS were evaluated by the log-rank test. The significance level in the statistical tests was set at α = 0.05 (two-tailed), and *p* < 0.05 was considered as being indicative of a statistically significant difference. Statistical analyses were performed using EZR version 2.7-1 software (Saitama Medical Center, Jichi Medical University, Saitama, Japan) [29]. Multiple comparisons were not considered because of the exploratory nature of this study.

## 3. Results

### 3.1. Odds Ratio by Logistic Regression Analysis

As shown in Table 1, the histological types were DLBCL (NOS) in 28 cases (67%) and HGBCL in 14 cases (33%). The HGBCL was classified as a DHL in four cases (10%) and as MYC translocation-only in four cases (10%), including HIV in one case. In HGBL (NOS) without MYC translocation, there were four cases (10%) of transformation from follicular lymphoma (FL) and two cases (5%) of transformation from lymphoplasmacytic lymphoma (LPL). There were 28 cases (67%) that were classified as R-IPI-poor or advanced-stage disease and 29 cases (69%) with elevated serum LDH levels. The results of immunohistochemistry for 17 factors [4,5,6,7,8,9,10,11,12,13,14,15,16,17,18,19,20,21,22,23,24,25,26,27,28] involved in anticancer drug metabolism were determined using FFPE samples.

As shown in Table 1, after R-CHOP, 22 patients showed CR, including 16 who did not develop relapse and 6 who developed relapse, and 20 patients showed non-CR. The median OS and median PFS were 64 months and 29 months in these two groups, respectively.

Elevated odds ratios for non-remission or relapse relative to remission set as the control were determined for R-IPI-poor (odds ratio: 5.4, *p* < 0.05) and HGBCL (6.0, *p* < 0.05), as well as for cases showing positive tumor expressions of MDR1 (24.00, *p* < 0.001), MRP1 (9.37, *p* < 0.05), and AKR1C3 (5.56, *p* < 0.01), all of which were then identified as statistically significant poor prognostic factors. For reference, the odds ratios were 5.77 (*p* > 0.05) for tumors showing the expression of GRP94 and 2.20 (*p* > 0.05) for tumors showing p53 expression; the ratios for tumors showing CYP3A4 expression were not evaluable, because the calculation was not applicable.

### 3.2. Kaplan–Meier Survival Curves and Between-Group Comparisons (Log-Rank Test)

Table 2 shows the median cumulative survival rates and times in the 42 LBCL patients determined by the Kaplan–Meier method and the results of between-group comparisons (*p*-value: log-rank test). Differences in survival in relation to poor prognostic factors were evaluated. The following were identified as statistically significant (*p* < 0.05) poor prognostic factors: tumor expressions of GRP94, TGFβ1, AKR1C3, and CYP3A4 and tumors classified as HGBCL, as indicated with (#). In addition, statistically significant combinations of poor prognostic factors were MRP1 or p53, AKR1C3+ or MDR1+, and GRP94+ and CYP3A4+. For the “AKR1C3 or MDR1” factor, the detail comments in Table 2 are as follows ((1)–(4)):

(1) Four endoplasmic reticulum stress (ER) proteins; (2) three metabolic enzymes of anticancer drugs; (3) three types of anticancer drug efflux pumps; (4) other proteins (three prognostic indices and seven other proteins).

(1) As ER stress proteins, the following four important proteins were selected: GRP94 [4,5,6], GRP78 [7,8], TGFβ1 [9,10], and TNFα1 [11]. GRP94 and GRP78 are stress-inducible molecules released into the extracellular space that act to overcome various stresses in the tumor microenvironment, including hypoxia, hypoglycemia, dysregulation of homeostasis, altered cellular metabolism, and acidosis. TGFβ1 plays an important role in promoting tumor progression. TNF inhibits tumor progression.
(2) As enzymes involved in anticancer drug metabolism, the following three enzymes were selected: AKR1C3 [12,13,14,15,16], CYP3A4) [17,18], and CYP2B6 [19]. AKR1C3 lowers the activities of daunorubicin, hydroxyl doxorubicin (enzyme involved in the metabolism of H), idarubicin (by two- to five-fold), and oncovin (enzyme involved in the metabolism of O: vincristine) (enzyme involved in the metabolism of HO) [14]. Patients with treatment-resistant T-ALL were found to show tumor overexpression of AKR1C3 [16]. The risks of disease progression and death were elevated in patients with diffuse large B-cell lymphoma (DLBCL) carrying the CC genotype of AKR1C3 [12]. CYP3A4 inactivates many anticancer drugs. Therefore, drugs showing intratumoral distribution intra-tumoral drugs, such as PTCL, may be further inactivated. As a result, the efficacy of these drugs may be lowered, leading to the development of drug resistance [17,18]. CYP62B6 activates cyclophosphamide [19].
(3) As anticancer drug efflux pumps, the following three proteins were selected: MDR1 [20,21,22], MRP1 [23,24], and MRP4 [25]. MDR1 and MRP1, found on the cell membranes, are oncovin hydroxyl doxorubicin (OH) efflux pumps. Tumor overexpression of MDR1 and MRP1 leads to the development of drug resistance [20]. DLBCL patients with relatively low expression levels of MDR1 have a good prognosis [21].
(4) Other items (two prognostic indices and seven other proteins) include the revised International R-IPI-poor and HGBCL, including DHL, follicular lymphoma transformation, lymphoplasmacytic lymphoma transformation, and HIV-related Burkitt lymphoma. In addition, double-expression (expression of both MYC and BCL2), p53, Ki-67 [26], CD5, glutathione-S-transferase (GST) [27], presence/absence of fibrosis, and thymidine phosphate [28] were also investigated.

**Figure 1:**
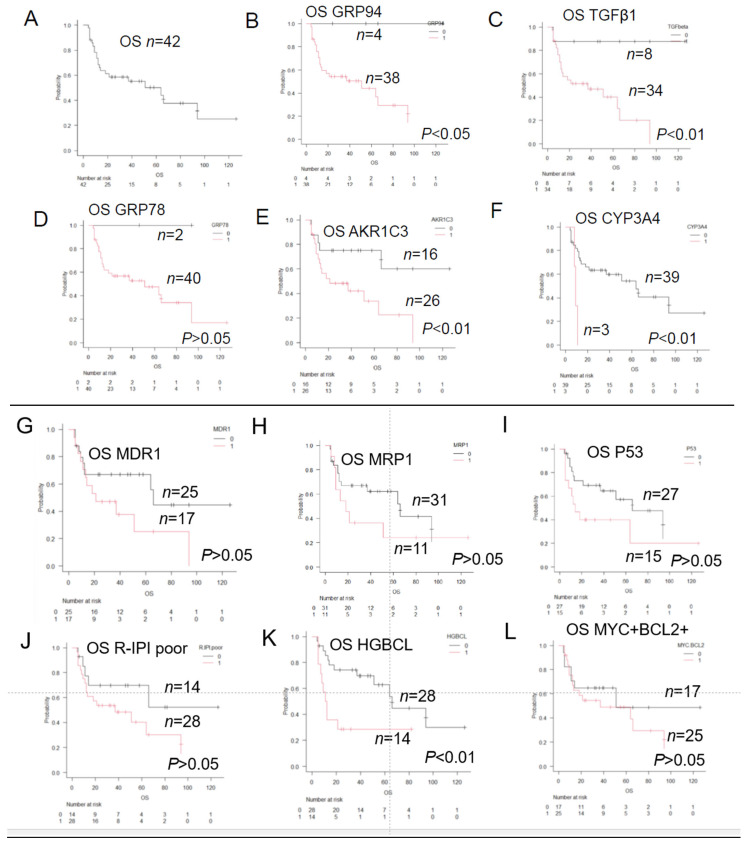

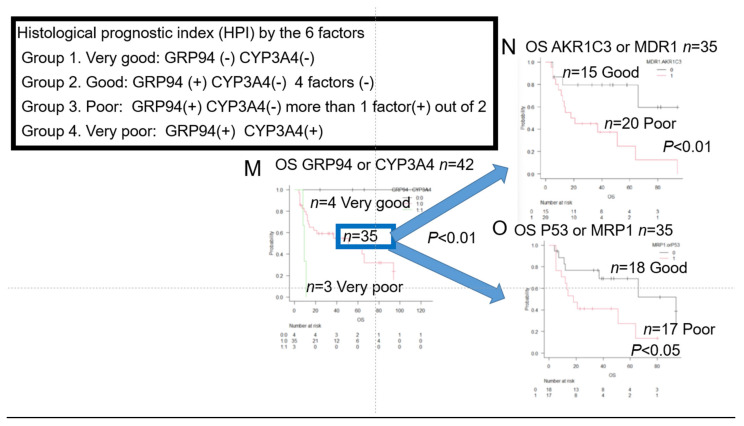
Overall survival of patients with and without the prognostic factors. Kaplan–Meier survival curves and between-group comparisons (log-rank test). (**A**) Overall survival (*n* = 42); (**B**) GRP94: negative, *n* = 4; positive, *n* = 38, *p* < 0.05; (**C**) TGF1: negative, *n* = 8; positive, *n* = 34, *p* < 0.05; (**D**) GRP78: negative, *n* = 2; positive, *n* = 40, *p* > 0.05; (**E**) AKR1C3: negative *n* = 16; positive, *n* = 26, *p* < 0.05; (**F**) CYP3A4: negative, *n* = 39; positive, *n* = 3, *p* < 0.05; (**G**) MDR1: negative, *n* = 25; positive, *n* = 17, *p* > 0.05; (**H**) MRP1: negative, *n* = 31; positive, *n* = 11, *p* > 0.05; (**I**) P53: negative, *n* = 27; positive, *n* = 15, *p* > 0.05; (**J**) R-IPI poor: negative *n* = 28; positive, *n* = 14, *p* > 0.05; (**K**) HGBCL: negative, *n* = 28; positive, *n* = 14, *p* > 0.05; (**L**) MYC+ BCL2+: negative *n* = 17; positive, *n* = 25, *p* > 0.05; (**M**) GRP94− CYP3A4−, *n* = 4 Group 1 Very good (5-year OS 100%), GRP94+ CYP3A4−, *n* = 35. Group 4 (Very poor, *n* = 3) consisted of 3 patients with tumor expression of both GRP94 and CYP3A4. All 3 patients died within a short period. *p* < 0.01; (**N**) AKR1C3 or MDR1: negative, *n* = 15; positive, *n* = 20, *p* < 0.01; (**O**) P53 or MRP1: negative, *n* = 18; positive, *n* = 17, *p* < 0.05.

The useful results were extracted from Table 2 and are presented in Figure 1. Single prognostic factors are listed as “A” and “K” in Figure 1. The combined prognostic factors are listed from “L” to “O” in Figure 1. In particular, the three-group comparison of survival rates and times according to the presence or absence of GRP94 and CYP3A4 expression shown in Figure 1M is important.

Group 1 (*n* = 4), the “Very good” group, consisted of four patients who showed negative staining for both GRP94 and CYP3A4, including two patients who were censored. This group had an extremely good prognosis, and all four patients survived (5-year OS: 100%). On the contrary, Group 4 (*n* = 3), the “Very poor” group, consisted of three patients who showed tumor expression of both GRP94 and CYP3A4. This group had a very poor prognosis, and all three patients died within a short period of time. The prognosis of the patients in Group 2 and Group 3 (*n* = 35) was intermediate, with a median survival of about 51 months. In Figure 1N,O, the intermediate prognosis group, Group 2 (*n* = 35), was subdivided into “Group 2 (Good)” consisting of patients who showed negative tumor IHC staining for both AKR1C3 and MDR1 and “Group 2 (Good)” consisting of patients who showed negative tumor IHC staining for both p53 and MRP1. The remaining patients included in Group 3 showed a poor prognosis.

Taken together, we would like to propose a new concept called the Histological Prognostic Index (HPI; Urayasu classification) as a predictor of the treatment response to R-CHOP in patients with new-onset primary LBCL. Based on the HPI, the patients included in this study could be classified into the following four prognostic groups: “Very good”, “Good”, “Poor”, and “Very poor”, according to the results of IHC staining for each of the six factors involved in LBCL: (1) Group 1 (“Very good” group) consisted of four patients who showed negative staining for both GRP94 and CYP3A4 and a 5-year OS of 100%; the four patients included two cases of DLBCL (NOS) classified as R-IPI-poor, one case of DLBCL (NOS) classified as R-IPI-good, and one case of DHL classified as R-IPI-good. (2) Group 2 (“Good” group) consisted of 15 or 18 patients who showed positive tumor staining for GRP94 and negative staining for CYP3A4 or negative staining for AKR1C3, MDR1, MRP1, and p53, with a 5-year OS of about 60–80% and median survival of 66–94 months. (3) Group 3 (“Poor” group) consisted of17 or 20 patients who showed positive tumor staining for GRP94, along with positive staining for one of the following four factors, namely, AKR1C3, MDR1, p53, and MRP1, with a 5-year OS of about 10–20% and median survival of 16–19.5 months. (4) Group 4 (“Very poor” group) consisted of 3 patients who showed positive tumor staining for CYP3A4, with a 1-year OS of 0% and medial survival of about 9 months. The breakdown of the three patients (Group 4) was as follows: one case of DLBCL (NOS) classified as R-IPI-good, one case of HGBCL (MYC translocation only) classified as R-IPI-poor, and one case of follicular lymphoma transformation classified as R-IPI-poor of the ER stress proteins other than GRP94; patients showing a positive tumor expression of TGF-beta1 (Figure 1C) and GRP78 (Figure 1D) may also be expected to have a good prognosis, as seen for the combinations (Table 2). Patients showing positive tumor staining for MDR1 or AKR1C3 showed a significantly poor prognosis (*p* < 0.01, Figure 1N), and even those classified into the “Good” group without R-IPI poor prognosis showed a poor prognosis. Also, of the patients with HGBCL, those who showed positive staining for MDR1 and AKR1C3 showed an even worse prognosis (*p* < 0.01). Of the patients with double-expression lymphomas (positive staining for both MYC and BCL2), those showing positive staining for MDR1 or AKR1C3 showed an even worse prognosis (*p* < 0.01). Five representative cases classified according to the immunohistochemical staining pattern and HPI.

#### Weighting of the Predictive Factors for the Survival Time

Weighting of the predictive factors for the survival time and combined multiple regression analysis yielded the following multiple regression equation (*p* < 0.05):

Predicted survival time (month) = −35.77 × GRP94 (0 or 1) + (−24.85 × (P53 or AKR1C3) (0 or 1)) + 86.39.

The effect is 0 if the IHC staining is negative and 1 if it is positive.

**Figure 2:**
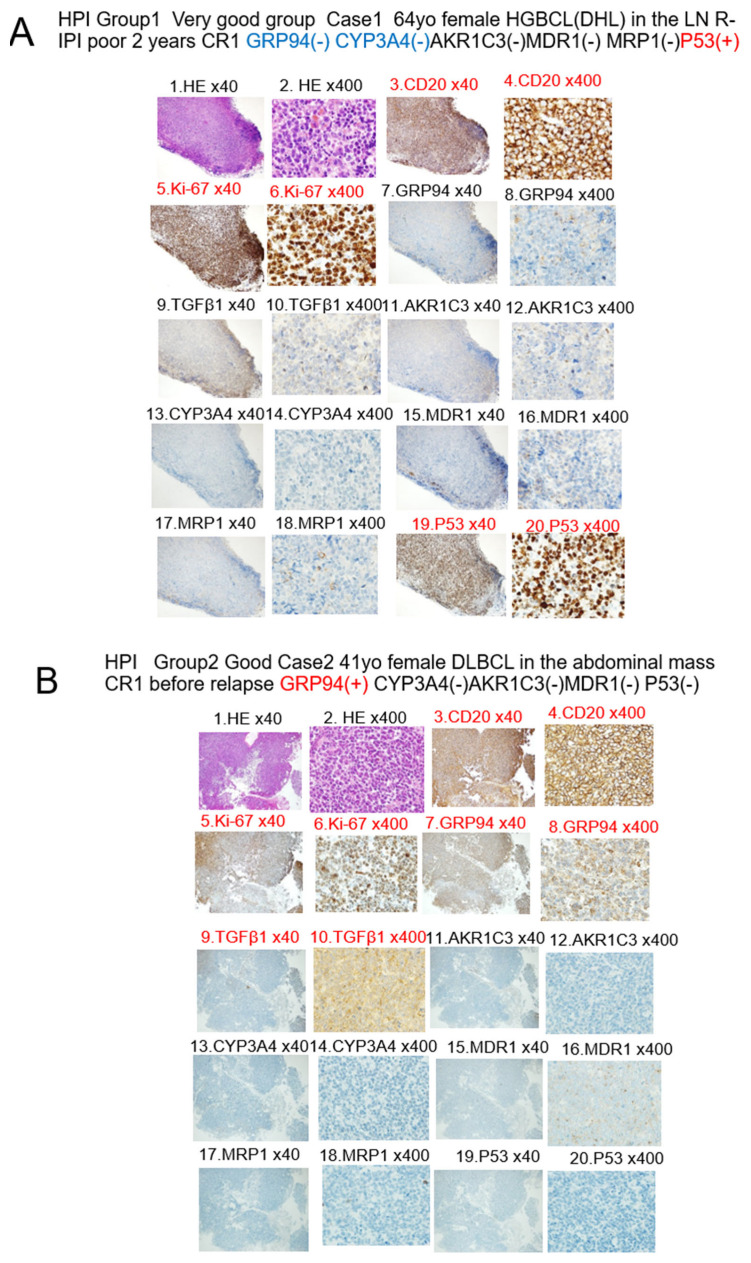

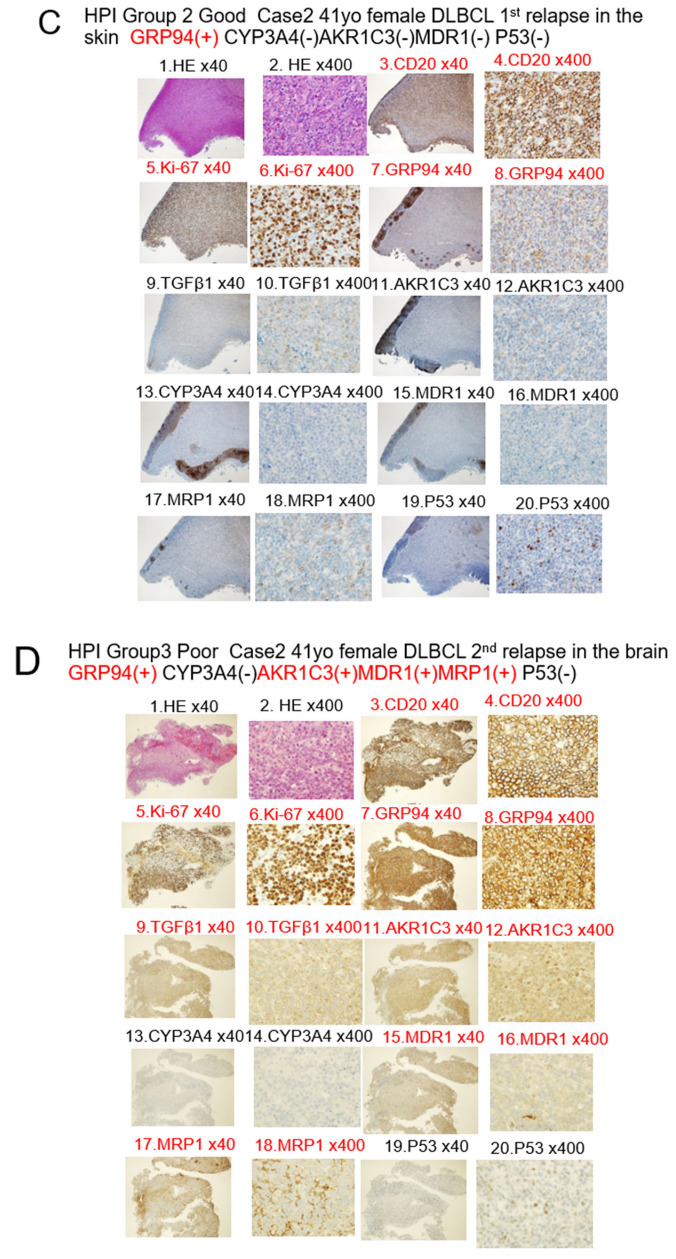

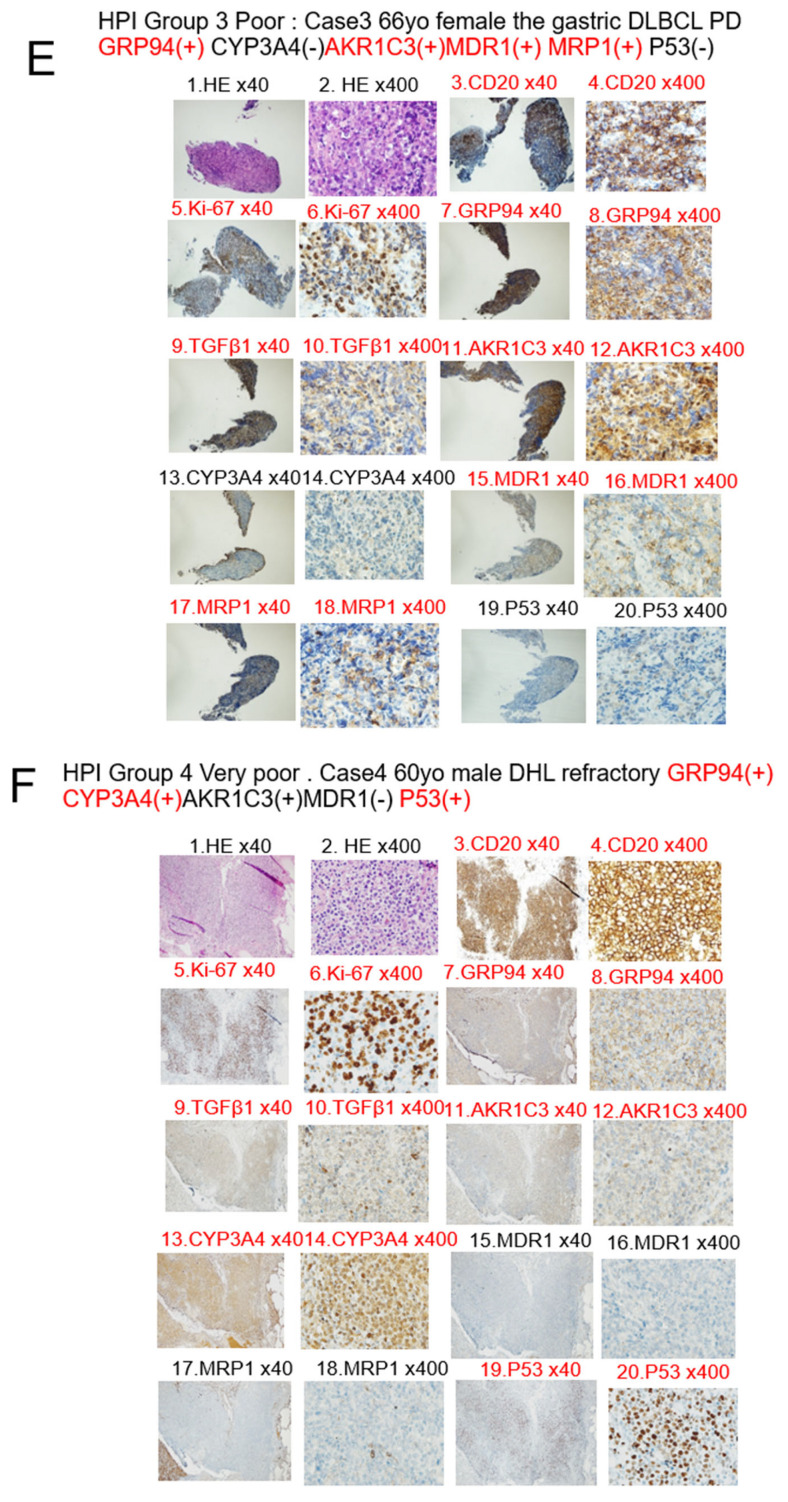

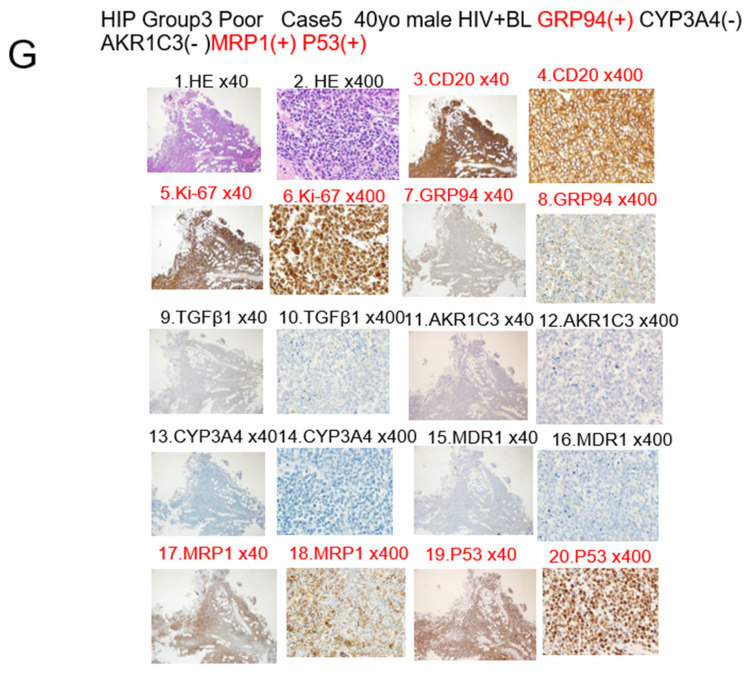
Five representative cases of the immunohistochemical staining patterns methods and HPI classification. Red indicates positivity, and black indicates negativity. CR1 indicates the first complete remission. HE indicates hematoxylin–eosin staining. (**A**) HPI-Group 1, “Very good”; Case 1: A 64-yo woman with HGBCL(DHL) in the lymph node. R-IPI-poor CR1 (2 years) GRP94(−) CYP3A4(−) AKR1C3(−) MDR1(−) MRP1(−) P53(+); (**B**) HPI-Group 2, “Good”; Case 2: A 41-yo woman with DLBCL in the LN CR1 before relapse; GRP94(+) CYP3A4(−) AKR1C3(−) MDR1(−) P53(−); (**C**) HPI-Group 2, “Good”; Case 2: A 41-yo woman with DLBCL 1st relapse in the skin; GRP94(+) CYP3A4(−) AKR1C3(−) MDR1(−); (**D**) HPI-Group 3, “Poor”; Case 2: A 41-yo woman with DLBCL 2nd relapse in the brain; GRP94(+) CYP3A4(−) AKR1C3(+) MDR1(+) P53(−); (**E**) HPI-Group 3, “Poor”; Case 3: A 66-yo female with gastric DLBCL; PD GRP94(+) CYP3A4(−) AKR1C3(+) MDR1(+) P53(−); (**F**) HPI-Group 4, “Very poor”; Case 4: A 60-yo man with refractory DHL; GRP94(+) CYP3A4(+) AKR1C3(+) MDR1(−) P53(+); (**G**) HPI-Group 3, “Poor”; Case 5: A 40-yo man with HIV + BL; GRP94(+) CYP3A4(−) AKR1C3(−) MRP1(+) P53(+).

Figure 2 shows cases classified by the HPI (Urayasu classification) IHC patterns.

2A: Case 1: A 64-year-old woman. She presented with enlarged retroperitoneal, mesenteric, and left cervical lymph nodes. Cervical biopsy revealed HGBCL (DHL), R-IPI-good. As shown in Figure 2A and Table 3, the tumor cells showed negative staining for GRP94, and the case was classified as HPI-Group 1 (“Very Good” group). After four cycles of R-CHOP therapy, the patient went into complete remission. Thereafter, she was found to have HGBCL (double-hit lymphoma), and CR has been maintained for about 2 years after three additional cycles of dose-adjusted etoposide, doxorubicin, and cyclophosphamide with vincristine, prednisone and rituximab (DA-EPOCH-R).

2B: Case 2: A 41-year-old woman. A biopsy of multiple peritoneal tumors revealed DLBCL (NOS), R-IPI-poor. As shown in Figure 2 and Table 3, the tumor cells showed positive IHC staining for GRP94 but negative staining for AKR1C3, MDR1, p53, and MRP1, and the patient was classified as HPI-Group 2 (“Good” group). After six cycles of R-CHOP therapy, CR was achieved. However, about one and a half years later, a biopsy of an enlarged lymph node in the leg revealed the first relapse.

2C: As shown in Figure 2C and Table 3, the tumor cells showed positive IHC staining for GRP94 and negative staining for the four factors (AKR1C3, MDR1, p53, and MRP1) and the case was classified as HPI-Group 2 (“Good” group). After R-ESHAP, autologous stem cell transplantation was performed, and a second CR was achieved. About 5 months later, cerebellar infiltration was detected, and a biopsy was performed, which revealed the second relapse.

2D: As shown in Figure 2D and Table 3, the tumor cells invading the cerebellum showed positive staining for GRP94 and three of the four factors (AKR1C3, MDR1, p53, and MRP1) and was classified as HPI-Group 3 (Poor). After four courses of high-dose methotrexate (MTX) + cytarabine (Ara-C) therapy, the third CR was achieved. About 9 months later, the patient developed a relapse in the central nervous system (CNS) and died.

2E: Case 3: A 66-year old woman. She was diagnosed as having primary gastric DLBCL (not otherwise specified (NOS)), R-IPI-poor. As shown in Figure 2E and Table 3, she showed positive tumor cell staining for GRP94 and three of the four factors (AKR1C3, MDR1, p53, and MRP1) and was classified as HPI-Group 3 (Poor). After eight cycles of R-CHOP therapy and two cycles of R-ESHAP therapy, the disease was found to be refractory. Autologous transplantation was performed after pretreatment with ranimustine, etoposide, cytarabine, and melphalan (MEAM). However, she developed relapse after radiotherapy and died.

2F: Case 4: A 60-year-old man. He had multiple enlarged lymph nodes around the abdominal aorta, mesentery, and bilateral iliac arteries, along with masses in the right lung, bilateral adrenal glands, and S7 of the liver. A lung biopsy revealed the diagnosis of HGBCL (MYC translocation only), R-IPI-poor. As shown in Figure 2F and Table 3, he showed positive tumor cell staining for both GRP94 and CYP3A4 and was classified as HPI-Group 4 (“Very poor” group). He received six cycles of R-CHOP therapy; two cycles of R-ESHAP therapy; IVAM (ifosfamide, etoposide, cytarabine, and methotrexate); and DeVIC (dexamethasone, etoposide, ifosfamide, and carboplatin). However, the disease proved refractory, and the patient died 1 year later.

2G: Case 5: A 40-year-old man. He was hospitalized for treatment of extramural obstruction of the common bile duct caused by mediastinal and hilar to para-aortic lymph node enlargement. A biopsy revealed the diagnosis of human immunodeficiency virus (HIV)-related BL (MYC translocation only), R-IPI-poor. As shown in Figure 2G and Table 3, he showed positive tumor cell staining for GRP94 and two of the four factors of AKR1C3, MDR1, p53, and MRP1 and was classified as HPI-Group 3 (“Poor” group). He did not respond to two cycles of CHOP; two cycles of DA-EPOCH-R; R-HDAC/MA (rituximab and high-dose cytarabine, with methotrexate and cytarabine); and ICE (ifosfamide, carboplatin, and etoposide). He died early, about 5 months after the start of treatment, due to CNS invasion and leukemic transformation. Table 3 summarizes the outcomes of the five cases.

## 4. Discussion

In general, under an optical microscope, LBCL cells can be seen infiltrating diffusely into adjacent tissues. It is relatively easy to identify LBCL with anti-CD20 antibodies. IHC staining is considered very useful, because it allows the assessment of positive and negative staining for various proteins while confirming the tumor cells. In this study, we conducted a retrospective IHC analysis for 17 proteins [4,5,6,7,8,9,10,11,12,13,14,15,16,17,18,19,20,21,22,23,24,25,26,27,28] that have been reported in the literature as being potentially important prognostic factors. Our results revealed that, of the 17 proteins, 6 (GRP94, CYP3A4, AKR1C3, MDR1, MRP1, and p53) were clinically significant.

ER stress proteins (GRP94, GRP78, and TGF-beta1) enable LBCL tumor cells to survive in harsh microenvironments. In the absence of ER stress proteins, the survival of LBCL tumor cells in harsh microenvironments becomes difficult, and the patients may be expected to survive for prolonged periods of time. The patients with LBCL, particularly all those who showed negative tumor staining for GRP94, survived (Figure 1B). Thus, patients showing negative tumor IHC staining for GRP94 were found to show an extremely good prognosis, and GRP94 could be a selective target for the treatment of this cancer [5,6].

The factors involved in the metabolism of the component anticancer drugs of the CHOP regimen promote the acquisition of resistance to antitumor drugs. The mechanisms include (1) decrease in drug uptake; (2) promotion of drug efflux; (3) improved DNA damage repair; (4) resistance to aging of cells (apoptosis); (5) changes in drug metabolism; (6) alterations in drug targets; (7) epigenetic modifications; (8) amplification of the target genes. These mechanisms, individually or in combination, lead to the development of the resistance of cancer cells to a single or multiple drugs [22].

Considering the above in slightly greater detail, “decrease in drug uptake” is unlikely to occur, because the intake of CHOP into cells is driven by a concentration gradient. However, equilibrative nucleoside transporter 1 (ENT1) is involved in the uptake of cytarabine and bendamastine [30]. In regard to “promotion of drug efflux”, MDR1 and MRP1 are particularly important (Figure 1N,O) [21]. As regards “improvement in DNA damage repair” and “resistance to aging of cells (apoptosis)” in malignant tumors, p53 mutations resulting in increased expression cause the loss of p53 function (Figure 1N,O) [26]. In regard to “changes in drug metabolism”, AKR1B1C3 is an enzyme involved in the metabolism of doxorubicin and vincristine [19]. In addition, CYP3A4 [17] is an enzyme involved in the metabolism of CHOP drugs (Figure 1E,F,M,N). For “alterations in drug targets”, “epigenetic modifications”, and “amplification of target genes”, please refer to other reports [2,3]. However, NOTCH1 [2], which is a poor prognostic marker, regulates the expression of MRP1 [31].

Of the ER stress proteins other than GRP94, patients showing positive tumor IHC staining for TGF-beta1 (Figure 1C) and GRP78- (Figure 1D) may also be expected to show a good prognosis. GST is a useful defense against reactive oxygen species induced by the H enzyme (reactive oxygen species induced by hydroxyl doxorubicin). GST inactivates the H enzyme in cooperation with the efflux pumps (MDR1 and MRP1) and AKR1C3, which inhibits the H enzyme [21]. Therefore, it is the expression of GST in patients showing positive tumor IHC staining for MDR1 or MRP1 or AKR1C3 that is considered to confer the poor prognosis (*p* < 0.05). On the other hand, since GST also inactivates bendamustine, it can be a reference for the selection of Pola BR (polatuzumab vedotin combined with bendamustine and rituximab) therapy in patients with relapsed/refractory LBCL.

As seen from the combinations shown in Table 2, even the “Good” group not classified as “RPI-poor” could have a poor prognosis. Also, among HGBCL patients, those showing positive results in IHC staining for MDR1 or AKR1C3 showed a worse prognosis (*p* < 0.01). Of the patients with double-expression lymphoma (positive results of IHC for both MYC and BCL2), those with positivity for MDR1 or AKR1C3 showed a worse prognosis (*p* < 0.01).

The results shown in Figure 2 are summarized in Table 3. HPI is more useful than R-IPI, because it takes into account the tumor resistance mechanisms and offers greater potential for the development of treatments stratified by the risk of treatment resistance.

In the future, the HPI (Urayasu classification) is expected to serve as a useful reference in clinical practice, along with HGBCL (Figure 1K), R-IPI, etc. The addition of IHC for six proteins at the time of the initial examination would allow classification by the HPI and the treatment stratified according to the pattern of tumor resistance treatment. For example, treatment selection can be made as follows: (1) for the “Very good” group, select R-CHOP; (2) for the “Good” group, select R-CHOP after the administration of a GRP94 inhibitor or anti-GRP94 antibody [4,5,6]; (3) for the “Poor” group, select R-CHOP after combination therapy using a GRP94 inhibitor, anti-GRP94 antibody [4,5,6], AKR1C3 inhibitor (epalrestat, a nonsteroidal anti-inflammatory drug (NSAID), etc.), or MDR1 and MRP1 inhibitor (cyclosporine A [32], etc.) [12,13,14,15,16]; and (4) for the “Very poor” group, select R-CHOP after the administration of a CYP3A4 inhibitor [17,18] or CYP3A4 inhibitor-conjugated anti-CD 20 antibody using a GRP94 inhibitor, an anti-GRP94 antibody [4,5,6]. When the therapy includes cytarabine, bendamustine, or gemcitabine, the use of ENT1, which regulates drug uptake, although not investigated in the present study, should be considered. If bendamustine is included in the therapy, the use of glutathione-S-transferase (GST) [27], a rate-limiting metabolic enzyme, may also be considered. In addition, it has been reported that NOTCH1, a marker of poor prognosis, regulates the expression of MRP1 [31]. In the future, it is expected that the recent findings [2,3] on clinically significant genes in LBCL will be integrated with the concept of the HPI.

It is, of course, acceptable to select Pola-R-CHP (polatuzumab with rituximab, cyclophosphamide, doxorubicin, and prednisone) therapy, instead of R-CHOP, for patients with new-onset advanced LBCL. Pola-R-CHP is considered as being essentially similar to R-CHOP in terms of metabolism, because Pola-R-CHP contains polatuzumab conjugated with monomethyl auristatin E (MMAE) in place of vincristine. In the case of HGBCL, intensive treatment regimens (DA-EPOCH; Hyper CVAD (hyperfractionated cyclophosphamide, vincristine, doxorubicin, and dexamethasone); etc.) with pretreatment can be selected instead of R-CHOP or Pola-R-CHP. This time, there was no publicly available database, and no dataset was available. This is an important issue that needs to be addressed in the future. We did not investigate copy number-related changes in GRP94 and CYP34A in the patient samples. We think this is an issue that we will investigate in the future.

For reference, after weighting the predictive factors for the survival time, a combined multiple regression analysis yielded the following multiple regression equation (*p* < 0.05):

Predicted survival time (month) = −35.77 × GRP94 (0 or 1) + (−24.85 × (P53 or AKR1C3) (0 or 1)) + 86.39.

The effect is 0 for negative IHC staining and 1 for positive IHC staining.

This prediction formula for predicting the survival prognosis is simple, practical, and useful for patients with LBCL. The most meaningful point about this prediction formula is that it is based on the treatment resistance mechanisms (1)–(3). More specifically, (1) the microenvironmental adaptability of the tumor cells was evaluated by IHC staining for GRP94; (2) resistance to OH, which is part of the CHOP therapeutic regimen, was evaluated by IHC staining for AKR1C3; and (3) an evaluation was also conducted for the tumor suppressor factor P53.

Our results suggested that the prognosis of cases with CYP3A4 positivity is exceptionally poor.

In summary, in patients diagnosed as having LBCL, IHC staining for the expression of at least six proteins (GRP94, CYP3A4, AKR1C3, p53, MDR1, and MRP1) should be performed at diagnosis for evaluation of the prognosis using the new HPI (Urayasu classification). If possible, testing should also be conducted for five additional molecules (TGF-beta1, GRP78, GST, MYC, and BCL2). Thus, testing for a total of 11 molecules, in addition to the conventional measurements, including for the identification of HGBCL (MYC and BCL2 translocations by FISH), R-IPI, and HIV antibody assay, would be desirable. Further studies are needed for patients with new-onset or relapsed LBCL using inhibitors and antibodies after evaluation of the HPI, including the development of other strategies to improve the outcomes of the treatment. The most meaningful point about this prediction formula is that it is based on the treatment resistance mechanisms. In the future, it is necessary to accumulate more cases for validating the findings of this study.

## Figures and Tables

**Table 1:** Characteristics of the LBCL patients included in this analysis (*n* = 42).

Characteristics of Patients in This Analysis	*n* = 42
Age > 60 years (%)	28 (67%)
Male (%)	25 (60%)
Histology	
DLBCL(NOS)	28 (67%)
GC	11 (26%)
non-GC	17 (40%)
CD5 positive (non-GC)	5 (12%)
HGBCL	14 (33%)
MYC and BCL2 translocation	4 (10%)
Only MYC translocation (including HIV *n* = 1)	4 (10%)
NOS (No MYC) with transformation	6 (14%)
from FL	4 (10%)
from LPL	2 (5%)
R-IPI	
0–2 very good plus good	14 (33%)
3–5 poor	28 (67%)
Stage	
Stage 1–2	14 (33%)
Stage 3–4	28 (67%)
Serum LDH	
Normal	13 (31%)
High	29 (69%)
R-CHOP outcome	
CR Non-relapse	16 (38%)
Relapse	6 (14%)
non-CR	20 (48%)
PD	14 (33%)
PR	6 (14%)
median OS (range)	64 M (4–126)
median PFS (range)	29 M (0–126)

Notes: Abbreviations: DLBCL, diffuse large B-cell lymphoma. NOS, not otherwise specified. HGBCL, high-grade B-cell lymphoma. R-IPI, revised International Prognostic Index. CR, complete remission. PD, progressive disease. PR, partial remission. OS, overall survival. PFS, progression-free survival.

**Table 2:** Summary of the immunohistochemical findings in cases of LBCL (*n* = 42).

Category	Factors (# Significant Difference)	*n*	Median OS (Months)	*p* Value	Figure 1	Reference
Total		42	64		A	
ER stress proteins	GRP94 (#)	38	51	* *p* < 0.05	B	[4,5,6]
	TGFβ1 (#)	34	37	** *p* < 0.01	C	[9,10]
	GRP78	40	51	*p* > 0.05	D	[7,8]
	TNFα1	24	24	*p* > 0.05		[11]
OH metabolic enzyme	AKR1C3 (#)	26	21	* *p* < 0.05	E	[12,13,14,15,16]
CHOP metabolic enzyme	CYP3A4 (#)	3	9	** *p* < 0.01	F	[17,18]
Cyclophosfamide activator	CYP2B6	19	94	*p* > 0.05		[19]
OH efflux pump	MDR1	17	21	*p* > 0.05	G	[20,21,22]
	MRP1	11	18	*p* > 0.05	H	[23,24]
MTX efflux pump	MRP4	2	5	*p* > 0.05		[25]
R-IPI	poor	28	37	*p* > 0.05	J	
HGBCL	DHL, Transformation, HIV (#)	14	11.5	** *p* < 0.01	K	
Double expression	MYC + BCL2 > 40%	25	37	*p* > 0.05	L	
Others	MYC > 40%	29	37	*p* > 0.05		
	P53 > 20%	15	14	*p* > 0.05	I	[26]
	CD5	5	18	*p* > 0.05		
	Ki-67 > 50%	40	64	*p* > 0.05		[26]
	GST	33	94	*p* > 0.05		[27]
	Fibrosis (Silver stain)	38	64	*p* > 0.05		[29]
	Thymidine phosphate backgrand	20	37	*p* > 0.05		[28]
Combination	AKR1C3+ or MDR1+ (#)	25	18	** *p* < 0.01	N	
	MRP1 or P53 (#)	22	16	* *p* < 0.05	O	[23,24,26]
	GRP94+ and CYP3A4+ (#)	3	9	** *p* < 0.01	M	
	GST (MDR1 or MRP1) (#)	19	37	* *p* < 0.05		
	GST (MDR1 or MRP1 or AKR1C3) (#)	26	51	* *p* < 0.05		
	R-IPI no poor (AKR1C3+ or MDR1+) (#)	4	9	* *p* < 0.05		
	HGBCL (CYP3A4+ or GRP94+) (#)	11	11	* *p* < 0.05		
	HGBCL (AKR1C3+ or MDR1+) (#)	9	11	** *p* < 0.01		
	MYC + BCL2+ (AKR1C3 or MDR1) (#)	15	13	** *p* < 0.01		

Notes: Among the many proteins expressed in cases of LBCL reported in the literature, we selected 17 proteins that we considered as being potentially important, along with 3 prognostic indices. Using antibodies against these proteins, a comprehensive retrospective immunohistochemical analysis of cases of LBCL was performed. The analysis was performed for the following 17 proteins and 3 prognostic indices: (1) 4 endoplasmic reticulum stress (ER) proteins; (2) 3 metabolic enzymes of anticancer drugs; (3) 3 types of anticancer drug efflux pumps; (4) others (3 prognostic indexes and 7 other factors). Abbreviations: OH, oncovin + hydroxyl doxorubicin. MTX, methotrexate sodium. DHL, double-hit lymphoma. #, *, ** all mean that there is a statistically significant difference.

**Table 3:** Summary of the outcomes of the five cases in Figure 2.

Figure 2	Case	Age	Disease	Characteristics	R-IPI	HPI (Nitta)	HPI 2 Factors		HPI 4 Factors				Treatment	Outcome	OS
		Sex	Figure 1K	Immuno-histostaing	Figure 1J	Figure 1M–O	GRP94	CYP3A4	AKR1C3	MDR1	MRP1	P53			months
A	Case 1	64 F	HGBCL	Onset	Poor	Group 1	(−)	(−)	(−)	(−)	(−)	(+)	R-CHOPx4	CR1	82
			DHL	GRP94(−)		Very good							DA-EPOCHRx3	Alive	
B	Case 2-1	41 F	DLBCL	Onset Bulky mass	Poor	Group 2	(+)	(−)	(−)	(−)	(−)	(−)	R-CHOPx6	CR1 45 M	64
			NOS	GRP94(+) 4factors(−)		Good								relapse	
C	Case 2-2	44 F	DLBCL	1st relapse skin	Good	Group 2	(+)	(−)	(−)	(−)	(−)	(−)	R-ESHAP	CR2 15 M	39
			NOS	GRP94(+) 4factors(−)		Good							ASCT	relapse	
D	Case 2-3	46 F	DLBCL	2nd relapse CNS	Good	Group 3	(+)	(−)	(+)	(+)	(+)	(−)	HDMTX-HDACx4	Dead	24
			NOS	GRP94(+) 3factors(+)		Poor							WBRT Tirabrutinib Steroid		
E	Case 3	66 F	DLBCL	Onset	Poor	Group 3	(+)	(−)	(+)	(+)	(+)	(−)	R-CHOPx8 PR	Dead	18
			NOS	GRP94(+) 3factors(+)		Poor							R-ESHAPx2 ASCT RT		
F	Case 4	60 M	HGBCL	Onset	Good	Group 4	(+)	(+)	(+)	(−)	(−)	(+)	R-CHOPx6	Dead	11
			DHL	CYP3A4(+)		Very poor							R-ESHAPx2 IVAM DeVIC		
G	Case 5	40 M	HBGCL	Onset HIV(+)	Good	Group 3	(+)	(−)	(−)	(−)	(+)	(+)	R-CHOPx2	Dead	5
			BL	GRP94(+) 2factors(+)		Poor							DA-EPOCH-Rx2		

Notes: Abbreviations: F: female; M: male; HPI: Histological Prognostic Index; R-IPI: revised International Prognostic Index; GRP94: glucose-regulated protein 94; CYP3A4: cytochrome p450 3A4; AKR1C3: aldo-keto reductase family 1 member C3; MDR1: multidrug resistance protein 1; MRP1: multidrug resistance-associated protein 1; OS: overall survival; HGBCL: high-grade B-cell lymphoma; DHL: double-hit lymphoma; DA: dose adjust; CR1: first complete remission; CR2: second complete remission: ASCT: autologous stem cell transplantation; CNS: central nervous system; HDAC: high-dose arabinoside; HDMTX: high-dose methotrexate; WBRT: whole-brain radiotherapy; PR: partial remission; RT: radiotherapy. (+): positive, (−): negative.

## Data Availability

The data presented in this study are available on request from the corresponding author. The data are not publicly available due to the problem of privacy issues.

## Abbreviations

AKR1C3: aldo-keto reductase family 1 member C3; Ara-C: cytarabine; ASCT: autologous stem cell transplantation; BCL2: B-cell lymphoma 2; CNS: central nervous system; CR: complete remission; CR1: first complete remission; CR2: second complete remission; CYP3A4: cytochrome P450 3A4; CYP2B6: cytochrome P450 2B6; DA: dose adjust; DHL: double-hit lymphoma; DLBCL (NOS): diffuse large B-cell lymphoma (not otherwise specified); ENT1: equilibrative nucleoside transporter 1; EPOCH: etoposide, Vincristine, Doxorubicin, Carboplatin, and Prednisolone; ER stress: endoplasmic reticulum stress; F: female; FFPE: formalin-fixed paraffin-embedded; FL: follicular lymphoma; GC: germinal center type; GRP78: glucose-regulated protein 78; GRP94: glucose-regulated protein 94; GST: glutathione-S-transferase; HGBCL: high-grade B-cell lymphoma; HDAC: high-dose arabinoside; HDMTX: high-dose methotrexate; HO: hydroxyl doxorubicin, oncovin (vincristine); HPI: Histological Prognostic Index; Hyper CVAD (hyperfractionated cyclophosphamide, vincristine, doxorubicin, and dexamethasone); IHC: immunohistochemical; LBCL: large B-cell lymphoma; LPL: lymphoplasmacytic lymphoma; MDR1: multidrug resistance protein 1; MMAE: monomethyl auristatin E; MRP1: multidrug resistance-associated protein 1; MRP4: multidrug resistance-associated protein 4; NOTCH1: notch receptor 1 transcription factor; NSAID: nonsteroidal anti-inflammatory drug; M: male; M: months; MTX: methotrexate sodium; OS, overall survival; PD: progressive disease; p53: P53 tumor suppressor transcription factor; PFS, progression-free survival; Pola R CHP (polatuzumab vedotin combined with cyclophosphamide, hydroxyl doxorubicin, and prednisolone; Pola BR (polatuzumab vedotin combined with bendamustine, rituximab, doxorubicin, and prednisolone); PR, partial remission; PTCL: peripheral T-cell lymphoma; R-CHOP: rituximab, cyclophosphamide, hydroxyl doxorubicin, oncovin (vincristine), prednisolone; R-IPI: revised International Prognostic Index; RT: radiotherapy; TGFβ1: transforming growth factor β1; NFα1: tumor necrosis factor α1; WBRT: whole-brain radiotherapy.
